# A Dental Congress

**Published:** 1885-10

**Authors:** 


### A DENTAL CONGRESS.

The editorial of last month, entitled "The Proposed International Dental Congress," is liable to convey a wrong impression upon one point. It was not, we believe, the intention of the mover of the resolution in the American Dental Association to suggest that a dental congress should be held at the same time and place with the proposed International Medical Congress. A meeting of the representative dentists of all nations, if it could be brought about, would undoubtedly prove an event of great importance to the profession, and might result in great good. If it cannot successfully be done through the Medical Congress, there is no reason why it might not be accomplished independently.

